# Outcome of self-expandable metal stents placement for obstructive colorectal cancer: 7 years’ experience from a Swedish tertiary center

**DOI:** 10.1007/s00464-022-09761-2

**Published:** 2022-11-18

**Authors:** Nikolaos Papachrysos, Morteza Shafazand, Leif Alkelin, Serta Kilincalp, Thomas de Lange

**Affiliations:** 1 Gastroenterology Section, Department of Medicine, Sahlgrenska University Hospital/Östra, Diagnosvägen 11, 416 50 Gothenburg, Sweden; 2 Department of Molecular and Clinical Medicine, Sahlgrenska Academy, University of Gothenburg, Gothenburg, Sweden; 3 Department of Medicine and Emergencies, Sahlgrenska University Hospital/Mölndal, Gothenburg, Sweden

**Keywords:** Colorectal cancer, SEMS, Large bowel obstruction, Endoscopy, Palliation, Surgery

## Abstract

**Background and study aims:**

Self expandable metal stents (SEMS) is an alternative to emergency surgery to treat malignant large bowel obstruction. It can be used either for palliation or as a bridge to curative surgery. Our study aims to review the outcomes of SEMS treatment in a tertiary center and to find predictors for the clinical outcome.

**Patient and methods:**

We retrospectively analyzed data from SEMS insertion at Sahlgrenska University Hospital, a referral center in Western Sweden (1.7 million inhabitants), between 2014 and 2020. Data collected were age, the intent of intervention, tumor localization, complication rate, technical and clinical success, 30- and 90-days mortality as well as long-term survival for the indication bridge to surgery.

**Results:**

We identified 265 SEMS insertions (mean age 72, female 49.4%). Most SEMS were used for palliation (90.2%). The malign obstruction was most often located in the left colon (71.7%). Technical success was achieved in 259 (97.7%) cases and clinical success in 244 (92.1%) cases. Post-operative complications occurred in 11 cases (4.2%). The 30-days mortality rate was 11.7% and the 90-day was 31.7%. In our analysis the tumor site was not associated with adverse outcomes and bridge to surgery indication was a positive prognostic factor for the 90-day mortality.

**Conclusions:**

We found that SEMS is an effective and safe treatment for patients with acute obstructive colorectal cancer.

Colorectal cancer (CRC) is one of the most common cancers with a global incidence of a million cases annually [1, 2]. In Sweden the incidence in 2018 was 50 per 100,000 inhabitants for women and 70 per 100,000 for men and caused 25–30 deaths/per 100,000 [3]. Approximately 8–20% of CRCs debut with acute obstructive symptoms [4–6]. Patients with obstructive CRC are usually older, have more advanced disease, more comorbidity, and are consequently at higher surgical risk [7]. Surgery for obstructive CRC is effective to relieve the obstruction either with intention of cure or as palliation with a permanent stoma but associated with high mortality and morbidity [8]. Endoscopic placement of self expanding metal stent (SEMS) has been used as an alternative to emergency surgery since the nineties. Even though the first endoscopically placed SEMS was reported by Dohmoto and Spinelli two decades ago [9, 10], the use of SEMS for palliation and as a bridge surgery has been assessed in several studies with various results [11–13]. Therefore, the exact role of SEMS remains unclear [14]. The latest ESGE guidelines from 2014 recommend SEMS only for palliation and not as a bridge to surgery [15]. However, in recent years, studies have not shown that SEMS as a bridge to surgery has poorer outcomes than surgery regarding clinical success, complications, and mortality. This fact still poses a question about the exact width of the indication of SEMS and makes the role of SEMS as a bridge to surgery controversial.

SEMS placement is a well-established method at our tertiary endoscopy center for several years. In this retrospective single-center study, we aim to analyze short- and long-term clinical outcomes of SEMS as palliation or bridge to surgery in patients with acute obstructive colorectal cancer and compare the outcomes for the two indications.

## Materials and methods

### Patient population

We performed a retrospective chart review to identify all patients that underwent placement of a colonic SEMS from January 2014 to December 2020 at Sahlgrenska University Hospital—Östra, a tertiary referral hospital for CRC in western Sweden (1.7 million inhabitants). Patients were identified through an administrative database that continuously registered all the SEMS insertions. The inclusion criteria were CT verified acute colonic obstruction or progressive obstructive symptoms in patients already diagnosed with colorectal cancer. Patients with SEMS insertion for a non-malignant cause or patients with extraluminal obstruction were excluded from the study. The obstruction was located from the right colon to the rectum. The purpose of stenting was either palliation or bridging to surgery.

From the electronic medical records, we collected demographic data including age, sex, location of the stenosis, operating endoscopist, intention (palliation vs bridge), and type of stent. The intention of stenting as bridge to surgery was determined post-hoc from the medical records and the decision of the multidisciplinary conference. The bridge to surgery stenting was followed-up by surgery with a curative intention (colectomy, liver resection, HIPEC/hyperthermic intraperitoneal chemotherapy). The following complications of stenting were recorded; perforation, stent migration, and bleeding. Patients were followed for a minimum of 3 months after SEMS insertion or until death.

For this study approval was obtained from the regional ethical committee in Gothenburg (Dnr 718-18/19-09-2018).

### Stent placement

All SEMS placements were performed under conscious sedation by endoscopists which had undergone specific training in SEMS placement and with extensive experience in therapeutic colonoscopy. All SEMS were through-the-scope TTS uncovered (WallFlex® colonic & WallFlex® Soft, Boston Scientific). They were placed under direct endoscopic and fluoroscopic visualization. Olympus CF-190Di endoscope were used to place the WallFlex colonic stents and Olympus PCF-190Di for the WallFlex Soft stent, a newer variant that only require an instrument canal of 9F/3.2 mm. After identifying the lesion, a hydrophilic guidewire (Hydra Jagwire, Boston Scientific) was inserted above the stricture within a cannulation catheter. The catheter was most commonly a 5.5 Fr ERCP cannulation catheter (Tandem XL, Boston Scientific) or replaced by a 4.4 Fr sphincterotome (Hydratome RX 44, Boston Scientific) if the access was difficult. A water-soluble contrast agent (Iohexol, Omnipaque®) was used to verify the exact position of the catheter, proximal to the obstruction and for estimating the obstruction length. Subsequently, stent deployment was performed over the guidewire by the through-the-scope TTS technique. Correct positioning was assured by fluoroscopy. The patients were prepared by one or two dosages of enema.

### Outcomes

Primary outcomes were technical and clinical success rate as well as complication rate. Our secondary outcomes were 30- and 90-days mortality data. We even tried to identify factors that could have an impact at procedure outcome.

#### Technical success

Technical success was defined as successful endoscopic placement of the stent that covered the entire length of the narrowed segment and proper expansion, verified by fluoroscopy.

#### Clinical success

Clinical success was defined as the ability to pass gas and stool with relief of the obstructive symptoms within 48 h, without the need for additional endoscopic intervention or surgery.

#### Complications

Complications were defined as perforation, bleeding, or stent migration.

### Statistical analysis

Descriptive statistics and odds ratios with 95% confidence limits were calculated. We used the chi-squared test or Fischer’s exact test to investigate associations between variables in groups with small observations. Statistical significance was considered as *p* < 0.05. Logistic regression was used to investigate risk factors for 30 and 90-day mortality and clinical success and adjusted for complications, elderly population at the procedure day (aged ≥ 75), and bridge to surgery intention. All data analysis was performed using SPSS Statistics for Windows version 28.0 (IBM SPSS).

## Results

### Baseline characteristics

We identified 265 SEMS procedures due to obstructive CRC in 234 patients. The average age of the patients was 72 years (range: 33–99). 134 patients (51%) were male. Most tumors were located in the left colon (72%). The intention for stent placement was palliation in 239 cases (90%) and as a bridge to surgery in 26 (10%). In 16 patients a second SEMS and in 5 patients a third SEMS were placed after tumor invasion and restenosis of the stent. The second SEMS were placed 2–36 months after the first one (Table 1).

**Table 1:** Baseline and oncologic characteristics of the 265 included cases

Mean age in years (range)	72 (33–99)
Gender F/M	131/134
Intention	
Palliation	239 (90.2%)
Bridge to surgery	26 (9.8%)
Obstruction location	
Rectum	15 (5.7%)
Left colon	190 (71.7%)
Transverse colon	28 (10.6%)
Right colon	32 (12.1%)

### Technical and clinical success

SEMS was successfully placed in 259 (97.7%) cases. 244 (92.1%) of the cases achieved symptomatic relief without the need for additional surgery or restenting within 30 days. If the result was clinically unsatisfactory colorectal surgeon determined the need for surgery (Table 2).

**Table 2:** Outcomes (*n* = 265)

Outcome	Number (%)
Technical success	259 (97.7)
Clinical success	244 (92.1)
Complications	11 (4.2)
Stent-related mortality	3 (1.1)
30 day mortality	31 (11.7)
90 day mortality	265 (31.7)

### Complications

Complications were rare. Eleven patients (4.2%) had a perforation during the first 48 postoperative hours and were immediately evaluated by a colorectal surgeon. No late perforations, nor stent migration or bleeding occurred during the 90-day follow-up period. Three of the perforations caused the patients’ death within few weeks after the SEMS insertion of whom one in the bridge to surgery group (Table 2).

### Mortality

Thirty-one patients (11.7%) died within 30 days after stent insertion and 84 patients (31.7%) died during the 90 days follow-up period. Except for the patients with stent perforation, no other stent-related mortality occurred. Most of the deaths were due to colorectal cancer progress. The SEMS related mortality were calculated at 1.1% (3/265).

### Palliative indication

Most patients received SEMS for palliation purposes to relief radiologically verified ileus or sub-ileus as an alternative to diverting stomia since several studies have shown that SEMS as palliation has important advantage to surgery since both overall quality of life (QoL) and QoL related to gastrointestinal symptoms is better [24–26].

The 30-day mortality in our palliation group was 12.6% (30/239). In nine cases a perforation occurred in our palliation group. Two of the patients had died because of the perforation and 4 patients died in total within 30 days from the SEMS insertion (range: 18–26 days) because of end stage cancer disease. The stent related mortality was calculated at 0.8% in the palliative group (2/239).

### Bridge to surgery stents

Thirteen women and thirteen men, mean age 64 (43–83) received SEMS as a bridge to surgery as decided at a Multidisciplinary Conference. The main reason was to avoid emergency surgery especially in the elderly patients with other comorbidities or in patients with metastatic disease in the waiting for the decision regarding potential curative surgery like hyperthermic intraperitoneal chemotherapy surgery (HIPEC) or liver surgery. In 15 patients, stenting was followed by colectomy, and in the remaining 11 it was followed by more extensive surgery, respectively colectomy & liver resection in 7, colectomy & HIPEC in 3, synchronous cancer in 1. In two cases the SEMS placement was complicated with perforation and one 82-year-old patient with obstruction in the left colon, suffering from comorbidities died some weeks after the stent insertion. 16/26 patients were alive until the second trimester of 2022. The death reason was mostly due to advanced disease and most of them were operated with HIPEC or synchronous liver resection. Three patients died in the colectomy group at an age of 79–82. Except for the patient with stent complication the other two patients died due to medical reasons and the death date was 6 years after the stent insertion. The 2-year mortality was 11.5% (3/26). The results in this subgroup are shown in Table 3.

**Table 3:** Outcomes in the bride to surgery group (*n* = 26)

Outcome	Number (%)
Obstruction site	
Rectum	1 (3.8)
Left colon	24 (92.3)
Transverse colon	1 (3.8)
Technical success	25 (96.2)
Clinical success	24 (92.3)
Complications	2 (7.7)
30-day mortality	1 (3.8)
90-day mortality	1 (3.8)
2 Year mortality	3 (11.5)

### Prognostic factors

We assess prognostic factors in a multiple logistic regression analysis and observed that only patients with complications had a statistically significant lower clinical success (*p* = 0.003, CI 95% 0.018–0.441). The tumor site was not associated with higher mortality or reduced clinical success. Clinical success was associated with a lower 30 and 90-day mortality. Bridge to surgery indication was a positive prognostic factor for 90-day mortality. Finally, 90-day mortality increases with age but the elderly population (defined as age ≥ 75) was no risk factor for either 30 or 90-day mortality (Table 4).

**Table 4:** Outcome after multiple logistic regression analysis—30 and 90-Day mortality

Covariates	30-day mortality		90-day mortality	
	OR (95% CI)	*p* Value	OR (95% CI)	*p* Value
Clinical success	0.152 (0.046–0.505)	0.002*	0.179 (0.054–0.593)	0.005*
Complications	5.156 (0.958–27.750)	0.056	3.284 (0.628–17.160)	0.159
Elderly population (≥ 75)	1.079 (0.244–4.779)	0.92	0.632 (0.241–1.661)	0.632
Age	1.029 (0.976–1.086)	0.29	1.038 (1.001–1.076)	0.041*
Bridge to surgery intention	0.185 (0.020–2.134)	0.19	0.054 (0.006–0.507)	0.011*

Data shown as odds ratio

*p < 0.05

## Discussion

This single-center study from a tertiary center is one of the largest published and demonstrates the importance and safety of SEMS placement in acute colorectal cancer obstruction. Nine out of ten patients had symptom relief with no need for emergency surgery and only less than one out of twenty developed complications.

Since 1990 few studies assessing SEMS for acute bowel obstruction are published and most of them have small sample size of less than 100 patients. The results from these studies are also conflicting with an important variation in the technical & clinical success as well as in the complication rate. Meisner et al. showed results close to ours in one of the largest international registry-based multicenter studies of 447 patients [16], with a technical success of 94.8%, clinical success of 90.5%, a complication rate of 3.4% (9 perforations, 6 stent migrations), and 30-day mortality at 8.9%. Another multicenter study from the UK that included 334 patients showed slightly lower technical success of 87.4%, clinical success of 83.5%, a complication rate of 14.8% among the palliative patients and 9.6% in the bridge to the surgery group, 30-day mortality was 13.6% in the palliative and 7.7% in the bridge to surgery group [17]. A recent multicenter study from Denmark of 239 patients with both bridge to surgery and palliative indication showed clinical success of only 81.6%, technical success 86.2% and a high rate of complications of 19.3% (perforation 8.8%, stent migration 10.5%) [18]. Their 30-day mortality was at 8.8%. Other smaller studies have a huge variation regarding the efficacy of SEMS with clinical success varying from 64 to 100% and a perforation rate from 3.6 to 10% [19, 20].

Meta-analyses from 2011 to 2014 comparing SEMS to emergency surgery did not show any statistical difference in postoperative mortality between the stent and surgery group but the SEMS group had much lower postoperative morbidity [21, 22]. This was despite that the SEMS groups had a lower success rate (76.9–79%) and a much higher complication rate (24–33%), than in our study. As a consequence and combined with concerns about less favorable oncological outcomes ESGE does not recommend SEMS as a bridge to surgery in acute bowel obstruction [15]. However, the conclusion may have changed with substantial higher success rate similar to ours in the SEMS group. Low volume studies included in the meta-analyses may have hampered the result since clinical success and complication rates is probably related to the endoscopist’s level of training and volume of procedures per endoscopist [15–26]. This may also apply to several prematurely stopped RCTs because of adverse events. Larger SEMS studies are more in accordance with our data with good efficacy and low adverse events rates when used as bridge to surgery [16, 17]. Recent studies have shown no difference in the oncological outcomes in the patients that get bridge-to-surgery stenting in comparison to emergency surgery in a follow-up period of 36 months [23].

We also found that the location of the obstruction doesn’t affect the outcome or the complications rate, and that makes SEMS insertion a good alternative even in malignant obstructions in the right colon. Our results even showed that elderly population did not have an increased rate of complications during SEMS insertion and that makes the SEMS alternative more attractive in this group because of the high perioperative morbidity during an acute operation.

SEMS as a palliative treatment in CRC has an important advantage to surgery since it both improves the patients’ overall quality of life (QoL) and the QoL related to gastrointestinal symptoms [24], and also improves calory intake [25]. Finally, SEMS is also better accepted than surgery by the patients [26].

The limitation of our study is the retrospective design and the inclusion of a single center. The main advantage of our study is the large number of patients included and the long follow-up. The excellent results may be explained by the fact that our center has more than 10 years of experience in colorectal SEMS, covers a population of about 1.7 million inhabitants and that all procedures are performed by experienced interventional endoscopists, thus a high volume per endoscopist and eventually a better technical success rate in comparison to a sporadic insertion of SEMS.

In summary, our results indicate that SEMS is a safe and effective procedure for patients with acute bowel obstruction secondary to CRC. It can also be used as a bridge to surgery for a later curative operation to decrease the need for emergency surgery and temporary stomia, but sufficiently powered randomized studies with at least 3 years of follow-up are needed to confirm the role of stenting as a bridge to surgery.
